# Caecal Volvulus in Pregnancy: A Rare Cause of Intestinal Obstruction and a Diagnostic Dilemma

**DOI:** 10.7759/cureus.97241

**Published:** 2025-11-19

**Authors:** Faria Rahman Antara, Anagha Vasant

**Affiliations:** 1 General Surgery, Ashford and St Peter's Hospitals NHS Foundation Trust, Chertsey, GBR; 2 Geriatrics, Ashford and St Peter's Hospitals NHS Foundation Trust, Chertsey, GBR

**Keywords:** abdominal pain, caecal volvulus, intestinal obstruction, pregnancy, right hemicolectomy

## Abstract

Caecal volvulus is an uncommon cause of intestinal obstruction during pregnancy. Despite advances in diagnostic imaging, the diagnosis of caecal volvulus remains challenging, as its presentation often resembles that of common pregnancy-related symptoms. Laboratory findings are often nonspecific and largely unremarkable due to physiological alterations associated with pregnancy. They tend to become abnormal when complications such as perforations or peritonitis occur, conditions that substantially increase maternal and fetal morbidity, and in severe cases, may result in fetal loss as reported in the literature. Hence, clinicians must maintain a high index of suspicion and promptly pursue appropriate imaging and management. We report a case of a 40-year-old woman at 27 weeks of gestation who presented with acute abdominal pain and constipation, diagnosed with caecal volvulus on magnetic resonance imaging (MRI) of the abdomen, and subsequently underwent a right hemicolectomy. Her pregnancy continued uneventfully, culminating in a healthy term delivery. This case emphasises the critical importance of maintaining high clinical suspicion and implementing timely multidisciplinary surgical management to achieve favourable maternal and fetal outcomes in this rare life-threatening condition.

## Introduction

Caecal volvulus, a torsion of the caecum, ascending colon and terminal ileum around their mesenteric axis, is a rare but serious cause of intestinal obstruction in pregnancy. It accounts for about 1-1.5% of intestinal obstruction in the general population and 25%-40% of colonic volvulus cases [1]. The occurrence of caecal volvulus during pregnancy is exceptionally rare, with an estimated incidence ranging from 1 in 1,500 to 1 in 66,000 deliveries, and only a limited number of cases have been documented in the literature [2].

Diagnosis in pregnancy is challenging due to anatomical displacement of bowel loops, and hesitancy to use radiological imaging, often delaying diagnosis and increasing maternal and foetal risk. Reported maternal mortality ranges from 6%-20%, while foetal mortality may reach 33% in cases of gangrenous bowel [3].

Surgical management options include detorsion, caecopexy, or right hemicolectomy, with the choice between primary anastomosis or stoma formation determined by intraoperative findings and the patient's overall condition. Herein, we present a case of a 40-year-old woman in her 27 weeks of gestation who was diagnosed with caecal volvulus after presenting with acute abdominal pain and underwent right hemicolectomy and ileostomy formation without interruption of pregnancy.

## Case presentation

A 40-year-old woman, Gravida 2 Para 1, at 27 weeks of gestation, presented with a 2-day history of progressive abdominal pain and a 1-day history of relative constipation. Her previous obstetric history included one uncomplicated spontaneous vaginal delivery, and the current pregnancy had been uneventful until this presentation. She had no significant past medical history apart from tonsillectomy at seven years of age, no previous abdominal surgeries, and no known drug allergies or regular medications aside from routine antenatal supplements. She denied any vaginal bleeding, discharge, or symptoms suggestive of preterm labour.

Examination findings

On examination, the patient appeared uncomfortable but hemodynamically stable. Vital signs were within normal limits. Abdominal examination revealed tenderness in the right upper quadrant with a soft, non-distended abdomen beyond that expected for a 27-week gravid uterus. There were no peritoneal signs initially. Obstetric examination confirmed a singleton pregnancy with palpable foetal movements, and cardiotocography demonstrated a normal foetal heart rate pattern with no evidence of uterine contractions. Transvaginal examination revealed a closed cervix with no evidence of premature rupture of membranes, vaginal bleeding, or discharge. The differential diagnoses considered included acute cholecystitis, appendicitis, intestinal obstruction and a general surgical review was sought.

Investigations

Initial laboratory findings are shown in Table 1.

**Table 1:** Initial laboratory findings on admission All biochemical values are expressed in standard SI units. mmol/L: millimoles per litre; µmol/L: micromoles per litre; mL/min/1.73 m²: millilitres per minute per 1.73 square meters of body surface area; mg/L: milligrams per litre; ×10⁹/L: cells per litre; g/L: grams per litre; fL: femtoliters

Parameter	Patient Value	Reference Range	Interpretation
Sodium (mmol/L)	136	135–145	Within normal limits
Potassium (mmol/L)	3.6	3.5–5.0	Within normal limits
Urea (mmol/L)	1.9	2.5–7.8	Slightly low (likely due to pregnancy-related changes)
Estimated Glomerular Filtration Rate (mL/min/1.73 m²)	>90	>90	Normal renal function
C-reactive Protein (mg/L)	13	<10	Mildly elevated
White Cell Count (×10⁹/L)	6.6	4.0–11.0	Normal
Haemoglobin (g/L)	117	115–155	Slightly low (physiological anaemia of pregnancy)
Platelets (×10⁹/L)	254	150–400	Normal
Mean Corpuscular Volume (fL)	96.2	80–100	Normal

Renal and liver function tests were unremarkable, and urinalysis was negative.

Transabdominal ultrasound examination demonstrated biliary sludge within the gallbladder, raising initial suspicion for acute cholecystitis in pregnancy, while the liver, kidneys, common bile duct, and spleen appeared normal. Transvaginal ultrasound excluded adnexal masses or cysts, free fluid, or collection in the right iliac fossa, and showed no evidence of acute appendicitis. Obstetric ultrasound confirmed a viable singleton intrauterine pregnancy with normal foetal cardiac activity, appropriate biometry for gestational age, adequate amniotic fluid, and a closed cervix.

**Figure 1:**
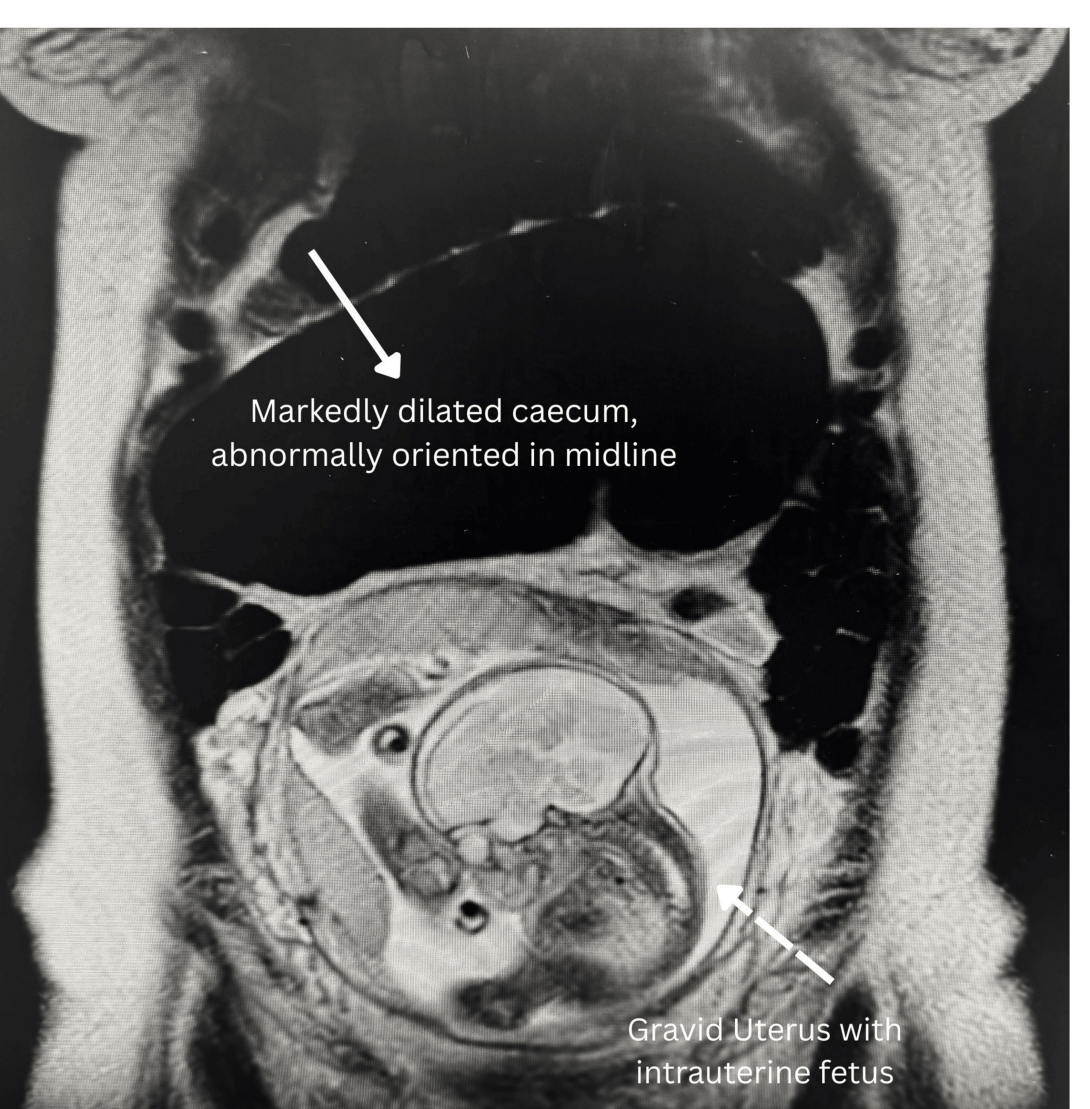
T2-weighted coronal magnetic resonance imaging (MRI) of the abdomen demonstrating a markedly dilated, abnormally oriented cecum positioned in the midline, consistent with caecal volvulus( solid white arrowhead). The gravid uterus with an intrauterine foetus is visualised inferiorly(broken arrowhead). MRI: magnetic resonance imaging

Given persistent abdominal pain despite normal initial imaging and serial examinations demonstrating absent bowel sounds, magnetic resonance imaging (MRI) of the abdomen was performed the following day. MRI findings revealed abnormal midline orientation of a dilated cecum with a transition point in the ascending colon, partial decompression of fluid into the terminal ileum with mild small bowel distension, and relative collapse of the colon distal to the ascending segment. The appendix was difficult to visualise due to caecal displacement, but no appreciable inflammatory changes were noted around the caecal pole. Small volume ascites was present within the right paracolic gutter. The radiological impression was consistent with caecal volvulus, although imaging suggested incomplete obstruction, given some decompression into the small bowel (Figure 1).

Management and surgical intervention

Following confirmation of the diagnosis, the patient was made nil by mouth, commenced on intravenous fluid resuscitation and broad-spectrum intravenous antibiotics. A multidisciplinary discussion involving general surgeons, obstetricians, anaesthetists, Intensive care specialists and the patient and her family resulted in a joint decision to proceed with emergency laparotomy, given the high risk of bowel ischemia and maternal-foetal complications.

The following day, the patient underwent emergency laparotomy. She was positioned supine with left lateral tilt to minimise aortocaval compression, a urinary catheter was inserted, and prophylactic intravenous antibiotics were administered. Intraoperative findings revealed a complete caecal volvulus with a grossly distended caecum demonstrating serosal tears and displacement into the epigastrium, dilated small bowel loops, and a gravid uterus appropriate for gestational age. Following detorsion of the volvulus, right hemicolectomy and ileostomy were performed.

The patient was transferred to the intensive care unit for close monitoring postoperatively. Obstetric ultrasound in the recovery unit was satisfactory, with continued foetal viability. Elevated inflammatory markers persisted in the early postoperative period. Table 2 summarises laboratory findings recorded on postoperative day two.

**Table 2:** Laboratory findings on postoperative day two All biochemical values are expressed in standard SI units. mmol/L: millimoles per litre; µmol/L: micromoles per litre; mL/min/1.73 m²: millilitres per minute per 1.73 square meters of body surface area; mg/L: milligrams per litre; ×10⁹/L: cells per litre; g/L: grams per litre; fL: femtoliters

Parameter	Result	Normal Range	Interpretation
Sodium (mmol/L)	138	135–145	Within normal limits
Potassium (mmol/L)	4.8	3.5–5.1	Normal
Urea (mmol/L)	1.9	2.5–7.1	Slightly low, not clinically significant
Creatinine (µmol/L)	42	44–80	Mildly low (reflective of pregnancy physiology)
Estimated Glomerular Filtration Rate (mL/min/1.73 m²)	>90	>90	Normal renal function
C-reactive Protein (mg/L)	153	<10	Elevated, consistent with postoperative inflammation
White Cell Count (×10⁹/L)	11.9	4.0–11.0	Mild leucocytosis, likely inflammatory
Haemoglobin (g/L)	110	120–160	Mild anaemia, expected postoperatively
Platelets (×10⁹/L)	336	150–400	Normal
Mean Corpuscular Volume (fL)	96.7	80–100	Normocytic

The patient developed postoperative ileus during the early postoperative period, which was managed conservatively with nasogastric tube decompression and total parenteral nutrition. She was subsequently stepped down to the labour ward from the intensive care unit due to concerns about possible premature rupture of membranes, as an ultrasound demonstrated oligohydramnios and raised inflammatory markers. Antenatal corticosteroids for foetal lung maturation were administered, and prophylactic oral erythromycin was commenced. Ileostomy function was established on postoperative day eight, and the patient was discharged home on postoperative day ten with outpatient obstetric and surgical follow-up arranged.

The pregnancy continued uneventfully to term, and the patient delivered a healthy baby via spontaneous vaginal delivery at full term. Both mother and baby were discharged in good condition, with plans for elective ileostomy reversal following completion of the postpartum period.

## Discussion

Caecal volvulus refers to a twisting, typically around 360 degrees, of the caecum and adjoining colon around their mesenteric axis. This rotation results in luminal obstruction at both ends of the affected segment and impairs mesenteric blood flow, producing a closed-loop obstruction and potential ischemia [4]. Caecal volvulus has two main types: axial rotation (90% of cases), in which the cecum twists counter-clockwise around the ileocolic vessels with involvement of the ileum, and caecal bascule, where the caecum folds upward and anteriorly in the horizontal plane, without torsion [1,4]. In our patient, intraoperative findings revealed a complete caecal volvulus with a grossly distended caecum, serosal tears, and displacement of the loop into the epigastric region, consistent with the axial rotation variant.

Multiple risk factors have been described for the development of caecal volvulus, including chronic constipation, laxative abuse, pregnancy, Chagas disease, previous abdominal surgery, adhesions, prolonged immobility, and anatomical variations such as a congenitally mobile caecum with incomplete peritoneal fixation [4,5]. The incidence of caecal volvulus during pregnancy increases with gestational age and is greatest at times of rapid uterine size changes, particularly between 16 and 20 weeks when the uterus becomes an intra-abdominal organ, from 32-36 weeks as the foetus descends into the pelvis, and in the puerperium when uterine dimensions change rapidly again [6]. Our patient, at 27 weeks of gestation, possessed several key predisposing factors: physiological changes during pregnancy and adhesions with scarring near the proximal transverse colon noted during operation.

The most common presenting symptoms in caecal volvulus include abdominal pain (98.8%), vomiting (61.5%), obstipation (30.4%), and nausea (25.4%), which are frequently mistaken for hyperemesis gravidarum, dyspepsia, cholecystitis, or preeclampsia, particularly problematic since nausea and vomiting occur in approximately 50% of pregnant women during the first trimester [3,7]. Our patient presented with acute abdominal pain, right upper quadrant tenderness, and a one-day history of constipation, symptoms that could easily be attributed to common pregnancy-related complaints, thereby underscoring the critical importance of maintaining high clinical suspicion for surgical emergencies and not hesitating to pursue appropriate diagnostic imaging despite concerns about foetal radiation exposure. Our case also confirms this diagnostic dilemma, as an MRI abdomen was performed for persistent pain and absent bowel sounds on serial abdominal examinations, leading to a diagnosis and surgery performed four days after symptom onset.

The diagnosis of caecal volvulus remains challenging and is often confirmed intraoperatively due to non-specific symptoms and limitations of imaging modalities. Though various imaging modalities offer varying degrees of diagnostic utility, clinical hesitancy to perform radiological investigations in pregnant patients represents a significant barrier to timely diagnosis. Typically, no single diagnostic imaging procedure delivers more than 50 miligray (mGy) of radiation, while guidelines recommend that the total cumulative foetal exposure during pregnancy should be kept below 50-100 mGy [8]. CT scanning can play a crucial role in diagnosing caecal volvulus, offering a sensitivity of up to 90% [4]. However, it exposes the foetus to radiation doses approaching 50 mGy [8]. In comparison, plain abdominal radiography offers comparable sensitivity of 95% with significantly lower foetal radiation exposure of only 1-3 mGy [5,8].

Abdominal ultrasonography remains a non-invasive, readily accessible, and radiation-free imaging modality applicable in suspected caecal volvulus. The characteristic “whirlpool sign” - representing the coiling of the superior mesenteric vein (SMV), mesenteric fat and associated bowel loops as they wrap around the superior mesenteric artery (SMA) - can assist in diagnosing midgut malrotation and volvulus; however, it is not specific for caecal volvulus on ultrasound [5]. Additionally, ultrasound serves as an invaluable tool for detecting free peritoneal fluid and excluding alternative obstetric and non-obstetric acute abdominal pathologies, such as acute cholecystitis, acute appendicitis, ovarian pathologies, which is particularly important in pregnant patients, where differential diagnosis can be challenging. In our case, transabdominal ultrasonography revealed biliary sludge in the gallbladder, raising initial suspicion for acute cholecystitis, while transvaginal ultrasound proved inconclusive, ultimately necessitating advanced imaging with MRI to establish the diagnosis.

Magnetic resonance imaging (MRI) offers superior multiplanar imaging capabilities with excellent soft tissue contrast while eliminating ionising radiation exposure, making it an ideal diagnostic tool for intestinal obstruction in pregnancy [9]. However, its clinical application is limited by high costs, requirement for specialised technical expertise, lengthy acquisition times, and restricted availability in emergency settings. In our case, given persistent symptoms despite initial ultrasound findings, MRI abdomen was performed and revealed abnormal midline orientation of a dilated cecum with a transition point in the ascending colon, partial decompression of fluid into the terminal ileum, mild small bowel distension, and relative collapse of the colon distal to the ascending segment, findings consistent with caecal volvulus that prompted urgent surgical intervention.

Treatment of caecal volvulus requires urgent surgical intervention in most cases. Surgical management encompasses various techniques, primarily dependent on bowel viability, the patient’s clinical condition, the presence of peritoneal contamination, anticipated recurrence rates and morbidity risk [4,10]. Non-resection surgical techniques, while less invasive, carry significant limitations in the management of caecal volvulus during pregnancy. Cecopexy, which involves securing the detorsed caecum to the parietal peritoneum, is associated with mortality rates up to 18% and recurrence rates ranging from 10-30% [3]. Similarly, cecostomy, a procedure involving insertion of a decompression tube into the distended cecum, carries substantial risks of recurrence and tube site leakage [3]. Given that pregnancy itself contributes as a risk factor for caecal volvulus due to progressive displacement of the caecum by the enlarging gravid uterus, these non-resectional approaches are frequently employed in pregnant patients, as demonstrated in the literature review.

Right hemicolectomy is the preferred surgical technique reported in the literature, as it offers the most definitive treatment and markedly reduces recurrence rates. The choice between performing a primary anastomosis or creating a stoma depends on the patient's overall physiological status, intraoperative findings and degree of peritoneal contamination [1,4]. The presence of gangrenous bowel mandates immediate resection, as the mortality rate escalates up to 33% when necrotic bowel is encountered, underscoring the significance of timely surgical intervention and individualised surgical planning tailored to operative findings [11].

In our case, following intraoperative detorsion of the volvulus, right hemicolectomy was performed due to intraoperative findings of a grossly distended caecum with serosal tears and adhesions at the point of torsion in the proximal transverse colon. An ileostomy was created instead of performing primary anastomosis to reduce the risk of anastomotic leakage, which has been reported to occur in 6.4%-8.8% of right hemicolectomy cases [12]. Pregnancies complicated by sepsis are associated with significantly increased rates of adverse obstetric outcomes, including emergency Caesarean delivery, postpartum haemorrhage, preterm delivery, and both maternal and foetal mortality, thereby supporting our approach to avoid primary anastomosis in this high-risk clinical scenario [13].

## Conclusions

Caecal volvulus is a rare but life-threatening cause of intestinal obstruction during pregnancy, where physiological and anatomical changes can both predispose to and obscure the diagnosis. A high index of suspicion is essential when evaluating persistent abdominal pain in pregnant patients, as delayed diagnosis can result in bowel gangrene, maternal sepsis and foetal loss. The safest available imaging modalities should be utilised to expedite diagnosis, and timely surgical intervention, which can serve both diagnostic and therapeutic purposes, must be pursued promptly in any clinical deterioration and should not be delayed. Multidisciplinary management and prompt surgical intervention - most effectively right hemicolectomy - are key to optimising maternal and foetal outcomes. Our case demonstrates that with clinical vigilance, appropriate investigation and timely intervention, caecal volvulus in pregnancy can be successfully managed while preserving both maternal and foetal well-being.
